# Diverse drought responses and evidence for stress priming in glacier-fed stream bacteria

**DOI:** 10.3389/fmicb.2026.1898462

**Published:** 2026-08-05

**Authors:** David Touchette, Florian Baier, Grégoire Michoud, Hannes Peter, Tom Battin

**Affiliations:** River Ecosystems Laboratory, Alpine and Polar Environmental Research Center, School of Architecture, Civil and Environmental Engineering, École Polytechnique Fédérale de Lausanne, Sion, Switzerland

**Keywords:** bacterial strains, biofilm, drought, glacier-fed stream, stress priming

## Abstract

Microorganisms inhabiting dynamic ecosystems are repeatedly exposed to environmental fluctuations and disturbances which require physiological adaptations, often associated with fitness trade-offs. In glacier-fed streams, benthic biofilms, the dominant microbial lifeform, frequently experience environmental fluctuations, including drying and rewetting. However, the diversity of responses of glacier-fed stream biofilm to drought stress is not well understood. For instance, *stress priming*, where prior exposure to a stressor enhances performance during subsequent events, has not been documented in glacier-fed stream bacteria. Here, we investigated how bacterial strains from glacier-fed stream biofilm respond to recurrent drying and whether they exhibit stress priming. To test this, we exposed 22 bacterial strains to repeated short (6 h) drying-rewetting cycles to assess whether their growth improves after repeated exposures. We further tested stress priming by assessing their survival to longer droughts (24 h and 96 h), quantified their biofilm formation, and explored their genomic traits to evaluate whether these features explain the various drought responses. Most strains survived a single short drought, but often at the cost of prolonged lag phases and reduced growth rates. Repeated drought exposure led to divergent stress responses: several strains exhibited signs of stress priming by improving growth traits and/or gaining the ability to withstand prolonged drought only after prior exposure. These responses were largely independent of taxonomy or genomic potential, suggesting that regulatory mechanisms dominate drought adaptation in glacier-fed stream bacteria. Overall, our findings highlight the response diversity among glacier-fed stream biofilm bacteria and show that adaptation often comes at the cost of altered growth dynamics, a potential strategy to cope with the natural hydrological fluctuations in glacier-fed streams.

## Introduction

In natural ecosystems, microbial communities can experience both stable and fluctuating conditions. However, increased rates of environmental change are intensifying environmental disturbances and driving shifts in physicochemical parameters, such as temperature, nutrient availability, light, and moisture, thus challenging the capacity of ecosystems to maintain their functions (Philippot et al., 2021). These changes in environmental conditions often impose strong physiological challenges, forcing microorganisms to adopt specific stress adaptation strategies (Schimel et al., 2007). For example, marine microbial communities exhibit temporal niche partitioning for nitrogen uptake over the diel cycle (Muratore et al., 2022), glacier microorganisms respond to repetitive freeze–thaw cycles by entering and exiting dormancy (Bradley et al., 2023), streambed microbial biofilms increase their extracellular polymeric substances (EPS) to mitigate desiccation (Romaní et al., 2025), and estuarine microbes employ either “salt-in” or “salt-out” osmoregulatory strategies by importing inorganic ions or compatible solutes to withstand tidal salinity fluctuations (Wu et al., 2024).

At the community level, responses to environmental stress can be broadly categorized as resistance, resilience or sensitivity (Allison and Martiny, 2008; Shade et al., 2012). These concepts also apply to the population and individual levels, where microbes possess intrinsic capacities to resist or recover from stress. However, such adaptive abilities often come with a cost, reflecting a fitness trade-off between growth performance and stress tolerance or adaptability (Acerenza, 2016; Zhu and Dai, 2024). These trade-offs may arise from resource reallocation toward stress-adaptation mechanisms (Schimel et al., 2007; Ferenci, 2016) or from metabolic optimization that favors efficient growth (Basan, 2018). Thus, based on their response to environmental stress, microorganisms can be classified according to whether their growth traits are unchanged or improved under stress (resistant), temporarily affected but return to baseline (resilient), viable but permanently altered (tolerant), not able to overcome repeated exposure (susceptible), or simply non-viable (sensitive).

Beyond the conceptual framework of responses to environmental stress, increasing evidence suggests that many microorganisms can show an enhanced response to a subsequent stress exposure compared to the initial one, a phenomenon referred to as *stress priming*, which is part of the broader concept of *stress memory* (Andrade-Linares et al., 2016; Wesener and Tietjen, 2019). Such stress priming is linked to genetic, epigenetic and biochemical processes (Scanlon et al., 2025) and can arise through multiple mechanisms, inducing DNA methylation, histone modifications, activation of transcription factors, and population-level bet-hedging (Guha et al., 2025; Zakrzewska et al., 2011; Bonilla, 2020; Lambert and Kussell, 2014; Solopova et al., 2014). However, most of these studies have focused on model organisms (e.g., *S. cerevisiae*, *B. subtilis*, *E. coli*) or on soil microorganisms, while few have examined freshwater ecosystems (e.g., Mathis and Ackermann, 2016). Consequently, the ecological significance and prevalence of drought adaptation and stress priming in freshwater ecosystems remain poorly understood.

Glacier-fed streams (GFSs) are a striking example of environments where microbial communities endure marked environmental fluctuations. These dynamic ecosystems are dominated by benthic biofilms (Ezzat et al., 2025; Michoud et al., 2025) that cope with diurnal and seasonal variations in temperature, nutrient and light regimes (Uehlinger et al., 2010; Milner et al., 2017). Discharge in GFSs is also highly variable, causing contractions of the river network (Malard et al., 2006; Llanos-Paez et al., 2025) and thereby exposing biofilms to recurrent drying-rewetting cycles and desiccation stress. Moreover, climate change will likely increase the frequency of droughts in these ecosystems (Paillex et al., 2020; Brunner et al., 2023). Although droughts can stimulate heterotrophic metabolism in GFS biofilm upon rewetting (Touchette et al., 2026), they also alter their microbial community composition, favoring drought-adapted taxa (Touchette et al., 2025).

Therefore, to explore whether GFS microorganisms are adapted to recurrent droughts and capable of stress priming, we conducted a laboratory drought-exposure experiment in which 22 bacterial strains isolated from GFS biofilms were subjected to repeated cycles of drying and rewetting. Growth traits were monitored across these repeated disturbances and survival to longer drought was assessed to identify the GFS strains responses to drought stress and whether they exhibit stress priming potential. In addition, we examined the strains’ genomes to identify traits associated with drought adaptation and priming capacity. Given the frequency of drying and rewetting in GFS, we expected that most strains would withstand drought, with responses ranging from resistance to tolerance. We further anticipated that their growth traits would improve with repeated droughts through priming, reflecting adaptation to the naturally fluctuating hydrological conditions of their environment.

## Materials and methods

### Bacteria isolation and selection

Bacteria were isolated from GFS biofilms grown on clay tiles in experimental flume mesocosms (Touchette et al., 2025) located in the Val Ferret catchment (Swiss Alps; 45.906 N, 7.124 E; 1774 m above sea level), as described by Gonzalez Mateu et al. (2026). Briefly, over 190 bacterial isolates were obtained by plating biofilm slurries on full-strength or diluted R2A, LB media, nutrient agar media or an iron-rich media, incubating at either 20 °C or 4 °C, and repeatedly re-streaking until pure cultures were obtained. Pure strains are kept at −80 °C in 20% glycerol for preservation. For this study, selected 22 bacterial strains representing a broad taxonomic diversity among the most prevalent and rare taxa in GFSs (Gonzalez Mateu et al., 2026; Touchette et al., 2025) and found worldwide (Ezzat et al., 2025), aiming to capture a broad range of ecologically relevant taxa (Table 1 and Supplementary Table 1). The strains encompassed nine orders, that are commonly dominant in GFSs, across five bacterial phyla (*Pseudomonadota n* = 16; *Bacteroidota n* = 2; *Actinomycetota n* = 2; *Bacillota n* = 1, *Deinococcota n* = 1) and included the *Burkholderiales* (*n* = 9), *Sphingomonadales* (*n* = 2), *Pseudomonadales* (*n* = 2), and *Flavobacteriales* (*n* = 1) families. Altogether, the selection comprised 20 distinct genera (Table 1). Additionally, two pairs of GFS strains from the same genus but different species (*Arthrobacter* and *Comamonas*) were included to assess inter-genus differences in drought response. All strains were able to grow under identical laboratory conditions despite differences in their initial isolation conditions to allow for direct comparison of growth trait responses and drought tolerance across phylogenetically and ecologically diverse bacteria.

**Table 1:** Genomic features and baseline growth traits of GFS bacterial strains.

Strain#	Strain genus	Genome completeness (%)	Genome contamination (%)	Genome size (Mbp)	%GC	16S rRNA copies	*r* (20 °C)	*t*_mid_ (h)	Biofilm^1^	Isolation media, temperature^2^
1	*Iodobacter* sp.	100.00	0.89	4.74	49	11	0.76	7.92	No	R2A, 20 °C
2	*Sphingomonas* sp.	100.00	0.24	4.26	65	4	0.45	9.65	Yes	$\frac{1}{50}$ R2A, 20 °C
3	*Arthrobacter* sp. 7	99.00	0.08	3.92	66	5	0.22	7.71	No	R2A, 30 °C
4	*Comamonas* sp.	100.00	0.01	3.61	59	7	0.40	11.06	No	NA, 20 °C
5	*Arthrobacter* sp. 45	99.94	0.03	4.88	66	5	0.31	6.21	No	$\frac{1}{10}$ NA, 20 °C
6	*Janthinobacterium* sp.	100.00	0.00	6.37	62	8	0.71	7.56	No	$\frac{1}{50}$ LB, 20 °C
7	*Comamonas acidovorans*	100.00	0.04	6.50	67	5	0.81	6.95	No	$\frac{1}{50}$ R2A, 30 °C
8	*Deinococcus aquaticus*	99.99	0.62	4.34	69	5	0.44	6.06	Yes	$\frac{1}{50}$ R2A, 30 °C
9	*Rahnella inusitata*	100.00	0.09	4.98	53	7	0.62	5.68	Yes	$\frac{1}{10}$ NA, 4 °C
10	*Telluria* sp.	100.00	0.20	5.36	64	7	0.60	8.50	No	$\frac{1}{10}$ NA, 4 °C
11	*Acinetobacter bohemicus*	92.28	6.09	3.96	40	7	0.67	5.77	Yes	$\frac{1}{10}$ R2A, 4 °C
12	*Klebsiella terrigena*	100.00	0.84	5.81	57	8	0.87	3.95	No	$\frac{1}{10}$ R2A, 30 °C
13	*Brevundimonas bullata*	99.99	0.05	3.38	67	3	0.34	7.44	Yes	SW, 20 °C
14	*Pedobacter gandavensis*	89.72	5.19	5.47	41	3	0.36	8.11	No	R2A, 4 °C
15	*Duganella* sp.	99.99	0.33	6.05	64	7	0.51	7.65	No	$\frac{1}{10}$ R2A, 4 °C
16	*Serratia proteamaculans*	100.00	0.13	5.61	55	7	0.71	5.41	Yes	R2A, 20 °C
17	*Acidovorax* sp.	100.00	1.42	7.02	67	4	0.28	13.32	No	SW, 4 °C
18	*Undibacterium undae*	98.96	0.00	4.42	45	4	0.46	18.30	No	SW, 4 °C
19	*Exiguobacterium* sp.	99.97	0.19	3.35	48	9	0.67	7.24	No	Fe, 20 °C
20	*Pseudomonas kulmbachensis*	100.00	0.12	5.15	58	8	0.56	4.59	No	SW, 4 °C
21	*Flavobacterium* sp.	100.00	0.00	5.52	36	5	0.63	6.48	No	Fe, 20 °C
22	*Rhodoferax* sp.	100.00	0.08	4.02	62	3	0.32	15.15	No	Fe, 20 °C

*t*_mid_: Time to reach half of the carrying capacity (lag-phase).

r: Growth rate.

^1^Baseline biofilm formation.

^2^R2A: Reasoner’s 2A, NA: nutrient agar, LB: lysogeny broth agar, SW: sterile stream water agar, Fe: iron-rich media agar. Data retrieved from Gonzalez Mateu et al. (2026).

### Drought-exposure experiment

**Figure 1:**
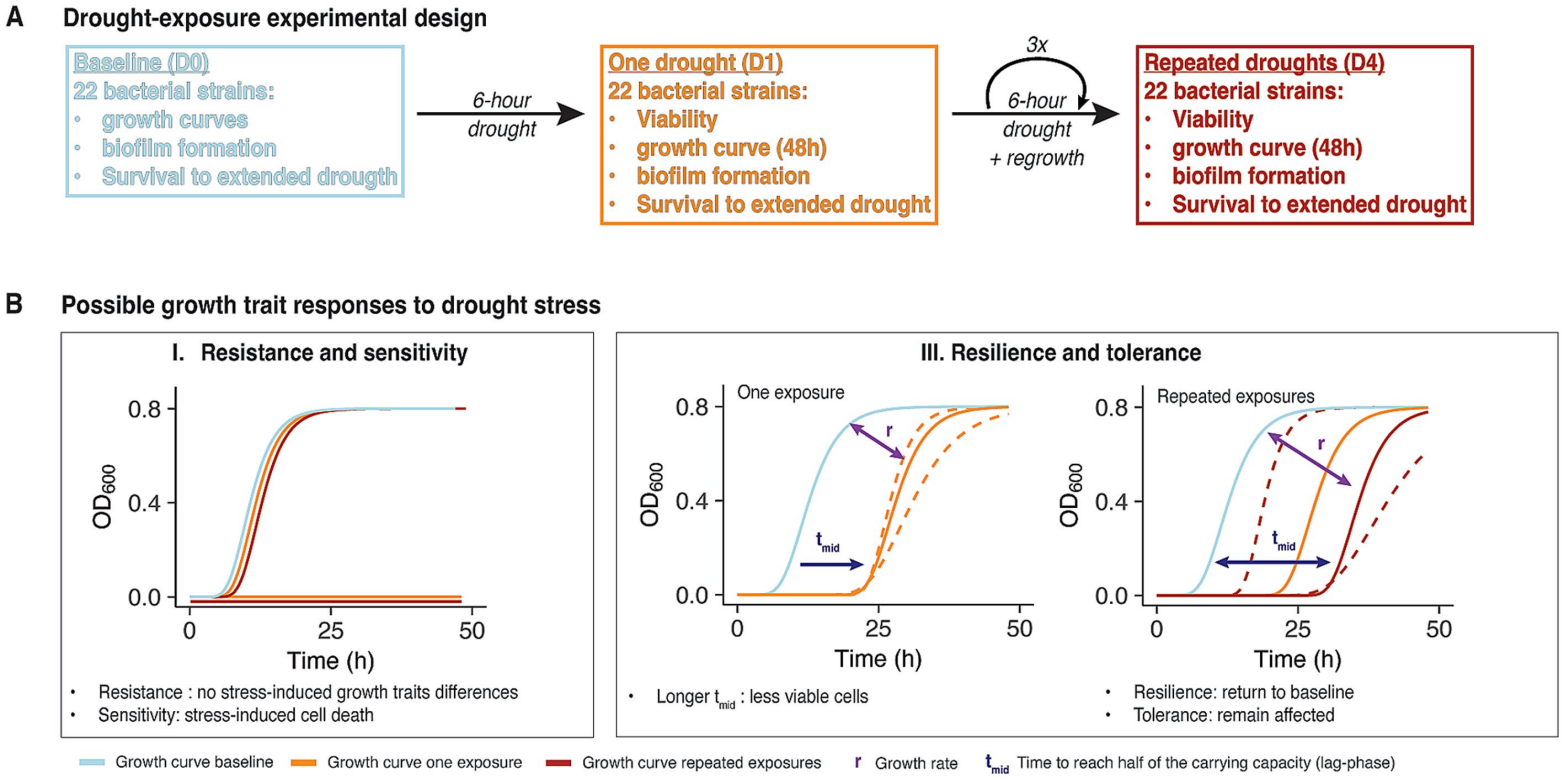
Drought-exposure experimental design and expected growth trait responses to drought stress. **(A)** Experimental design of the drought-exposure experiment on the 22 glacier-fed stream bacterial strains. **(B)** Possible induced growth trait changes by drought stress. Growth traits unchanged or improved: Resistance. Growth traits temporarily affected but returning to baseline: Resilience. Growth permanently altered but viable: Tolerance. Non-viable: Sensitivity. Arrows indicate the possible direction of growth traits (enhancement or impairment), following a single or repeated drought exposure.

To assess the drought response of the 22 GFS bacterial strains (Figure 1), initial cultures were prepared by inoculating four single colonies per strain (one per well), each picked from the same freshly streaked R2A agar plates prepared from −80 °C glycerol stocks, into 2 mL of R2A medium in a deep-well 96-well plate (Corning). This resulted in a plate layout containing four replicates per strain and eight uninoculated controls with R2A medium only. The plates were incubated for 48 h, after which 6 μL from each well was transferred to 194 μL of fresh R2A medium in a 96-well flat bottom microplate (Greiner Bio-One) and incubated for an additional 48 h. Plates used for desiccation were covered with Breathe-Easier sealing film (Diversified Biotech), while plates used for growth curve measurements were covered with a translucent Breathe-Easy film (Diversified Biotech). To facilitate laboratory work, all incubations were performed at 20 °C for 48 h on an orbital shaker (Huber Lab) set to 1,050 rpm, a biologically relevant temperature as the majority of GFS bacteria are mesophiles (Michoud et al., 2025), and most of the strains used here have an increased growth rate at high temperatures (Gonzalez Mateu et al., 2026).

Preliminary tests indicated that 12 h were required to achieve visible desiccation of 6 μL of R2A medium in 96-well microplates under our laboratory conditions (data not shown). After the initial 48 h growth period, 6 μL from each well was transferred into four new plates, respectively: two plates were set for a 6 h drought (18 h total drying time: 12 h until desiccation and 6 h drought); the other two plates received 194 μL of R2A, one to measure the baseline growth curve (D0) by monitoring OD_600_ every 30 min on a microplate reader (Synergy H1, Biotek) with continuous shaking and for biofilm formation assessment, and one used for survival to extended drought exposure. After the 6 h drought, strains in the desiccated plates were resuspended in 200 μL of R2A, where one plate was used to measure growth curve after one drought exposure (D1) and biofilm formation, and the other was reserved for viable cell quantification, extended drought survival, and repeated 6 h drought exposure. After a 48 h growth period, 6 μL of each well of the D1 plate was transferred to a new plate for another 6 h desiccation and subsequent resuspension as described above. This procedure was repeated for a total of four consecutive desiccation cycles of 6 h. After the fourth desiccation period, the resulting plates of strains were resuspended in 200 μL of R2A, where one plate was used to measure growth curves and biofilm formation after repeated drought exposure (D4), and the other was used for viable cell quantification and extended drought survival.

To assess strain tolerance to longer drought periods (Figure 1), 6 μL of secondary plates of D0, D1, and D4 treatments were transferred into new 96-well plates for either a 24 h drought (36 h total drying time: 12 h until desiccation and 24 h drought) or a 96 h drought (108 h total drying time: 12 h until desiccation and 96 h drought). After the desiccation period, strains were resuspended in 200 μL of R2A, and OD_600_ was measured immediately, and again after a 48 h incubation.

### Viable cell quantification

Viable cell numbers were assessed by plating out serially diluted samples on solid R2A agar medium. After each drought exposure, 200 μL of fresh sterile R2A medium was added to each well, and plates were incubated with gentle shaking (250 rpm) on an orbital shaker for 30 min. Strains were further resuspended by gentle repetitive pipetting, and 10 μL of the suspensions were transferred into 90 μL R2A. This 1:10 dilution step was repeated five times to obtain serial dilutions ranging from 10^−1^ to 10^−5^. Subsequently, 1 μL from each well and each dilution was plated on a R2A agar plate using a flame-sterilized replica plater. Plates were incubated at 20 °C for 78 h, after which the lowest dilution showing visible growth was used to calculate the initial number of viable cells per well (as colony forming units). We acknowledge that the use of a replica plater may affect the accuracy of viable cell quantification. However, this approach enabled the high-throughput processing and speed required for our experimental design.

### Biofilm formation assay

The same 96-well plates used for growth curve measurements were also used to assess biofilm formation by crystal violet staining (O’Toole, 2011) after a 48 h of growth following resuspension. The wells were emptied by plate inversion, washed three times with deionized water, and inverted to remove excess liquid. Each well was then filled with 0.01% (w/v) crystal violet solution and incubated on an orbital shaker for 30 min (250 rpm). The wells were subsequently washed three additional times with deionized water and air-dried completely in a laminar flow hood. The retained crystal violet was solubilized in 200 μL of 70% ethanol and incubated for 30 min on an orbital shaker before measuring absorbance at 590 nm (OD_590_).

### DNA extraction and whole-genome analysis

To sequence the genomes of the 22 bacterial strains, fresh cultures were inoculated from individual colonies into 5 mL of R2A medium and grown at 20 °C for 72 h. A 1.5 mL aliquot of each culture was pelleted and resuspended in 500 μL of DNA/RNA Shield (Zymo Research) supplemented with 50 μL of 20 mg/mL proteinase K (Zymo Research). The mixture was transferred into 1.5 mL tubes containing ~ 20% of 0.1 mm zirconium beads and vortexed at max speed for 30 min at 37 °C. DNA was then purified using the ZymoBIOMICS DNA Miniprep Kit (Zymo Research), according to the manufacturer’s instructions. DNA concentration and purity were assessed using the Qubit dsDNA HS Assay Kit (Invitrogen), and DNA size and integrity was confirmed with a Genomic DNA ScreenTape on a TapeStation system (Agilent). Libraries for long-read whole-genome sequencing were prepared using the Ligation Sequencing Kit and Native Barcoding Expansion 24 V14 (Oxford Nanopore Technologies), following the manufacturer’s protocol and using the recommended NEBNext reagents (New England Biolabs) for both ligation steps. A total of 400 ng of high-quality DNA per sample was used as input. Libraries were sequenced on a MinION Mk1B device using a R10.4.1 flow cell (ONT).

Raw sequences were basecalled, demultiplexed and trimmed using *dorado* v.0.8.0 (ONT) in “duplex sup” mode and were assembled using *Hybracter* v.0.11.2 (Bouras et al., 2024). Genome assemblies’ quality and completeness was assess with *CheckM2* v.1.0.2 (Chklovski et al., 2023), and their taxonomy was assign with *GTDB-Tk* v.2.2.2 (Chaumeil et al., 2022). Bacterial genomes were annotated with *mettannotator* v.1.4.0 (Gurbich et al., 2025), *Bakta* v.1.10.3 (Schwengers et al., 2021), and using the KEGG Orthology (KO) database via *eggNOG-mapper* v.2.1.12 (Cantalapiedra et al., 2021). The 16S rRNA gene copy number was identified with *Bakta*. A list of overall and osmotic/drought-specific stress tolerance genomic traits KEGG orthologs (KOs) was generated, including all KOs from the “Biofilm formation,” “Exopolysaccharide biosynthesis,” “Base excision repair,” “Nucleotide excision repair,” “Mismatch repair,” “Homologous recombination,” “DNA repair and recombination proteins,” “Lipopolysaccharide biosynthesis proteins,” “Peptidoglycan biosynthesis and degradation proteins,” “Chaperones and folding catalysts,” “Thermogenesis,” alongside temperature or drought specific “Transcription factors” and “Two-component system” KOs, as well as proline, glycine betaine, trehalose, ectoine, carnitine, osmoprotectants, sodium and potassium “ABC transporters,” “Transporters” and biosynthesis KOs. For each strain, the number of genes in these categories were normalized by dividing with the total number of genes in the genome. Strains overall genomic functional traits were identified with the microTrait pipeline v.1.0.0 (Karaoz and Brodie, 2022). Functional gene counts were used to assess the genomic potential for drought response, although gene presence does not necessarily reflect gene expression or activity. Genome assemblies are available in NCBI under the BioProject ID PRJNA1171349.

### Data analysis and statistics

All data analyses and visualizations were performed in R v.4.4.2 (R Core Team, 2024) using *ggplot2* v.3.5 (Wickham, 2016) and *pheatmap* v.1.0.13 (Kolde, 2025). Growth traits (r: growth rate; t_mid_: time to reach half of the carrying capacity, proxy for lag phase), were estimated from growth curves (D0, D1, D4) by logistic model fitting using the package *GrowthCurver* v.0.3.1 (Sprouffske and Wagner, 2016), with background correction (blank removal). Each growth curve was visually inspected, and one well from treatment D1 and five wells from treatment D4 showing abnormal behavior were excluded. Growth traits were re-estimated without these outliers (Supplementary Figure 1). Curves with carrying capacity <0.01 or with “cannot fit data” or “questionable fit” errors were considered as *no growth* (confirmed visually). Biofilm formation values (OD_590_) were normalized by subtracting the highest value of the blank R2A controls; negative values were set to zero. To avoid division by zero in downstream analysis, the lowest positive normalized OD_590_ value was added to denominators where required. Initial biofilm formation was considered positive if the average OD_590_ was at least 1.5 × the highest control value. Correlation between the number of genes associated with biofilm formation or extracellular polymeric substances (EPS) production and measured biofilm formation (mean OD_590_) was assessed with the “cor.test” function from the *stats* R v.4.4.2 package.

Differences in *r*, *t*_mid_, and biofilm formation between drought exposure treatments (D1, D4) and the baseline (D0) were tested using the Wilcoxon Rank Sum test, with corresponding alternative hypothesis, after checking normality of the data using the Shapiro–Wilk test, both from the R package *stats*. *p*-values were adjusted for multiple testing using the Benjamini–Hochberg (BH) method, and adjusted *p*-values < 0.05 were considered significantly different compared to the baseline. Log_2_ fold-changes were calculated as the ratio of the mean per-strain treatment value to the mean baseline value. For visualization purposes only, infinite or undefined *r* and *t*_mid_ values were replaced with fixed boundary values corresponding to the limits of the plotting range. These substitutions were not used for downstream statistical analyses. Survival of strains after extended drought exposure (24 h and 96 h) was evaluated by subtracting the initial OD_600_ value from the final OD_600_ value measured after 48 h of regrowth. Wells with a normalized OD_600_ value at least twice the highest blank R2A were considered to show growth. If the blank was ≤ 0, it was replaced by the smallest positive control value observed across all plates (0.001) to avoid a zero threshold. Strains with at least three out of four wells showing growth were considered to have survived the drought.

To assess the relationship between *t*_mid_ and cell viability after drought exposure, a linear model of the form *t*_mid_ ~ log(Viability) was fitted using the “lm” function from *stats*. For heatmap visualizations of growth trait changes, bacterial strains were clustered based on their changes in *r*, *t*_mid_, and biofilm formation, together with their survival outcome to extended drought. Clustering was performed using Euclidian distance calculated with the “vegdist” function from *vegan* v.2.6–10 (Oksanen et al., 2010) followed by hierarchical clustering implemented with “hclust” from *stats*. Differences in genomic traits among strains were explored using Bray–Curtis dissimilarities on the Hellinger-transformed microTrait granularity level 3 genomic feature matrix, transformed with “decostand” in *vegan*. Ordination was conducted by non-metric multidimensional scaling (NMDS) using the “metaMDS” function of *vegan* with 100 iterations. Effects of taxonomic and phenotypic factors “Order” and “Response category” on the overall genomic was tested with “adonis2” and 999 permutations, and the proportion of variation explained by each factor was quantified through variance partitioning using “varpart,” both functions implemented in *vegan*. For genomic functional visualization, “stress” genes count was normalized by the total number of genes per strain, and a z-transformation was applied by functional category. Codes and raw data used in this study are available on Github: RIVER-EPFL/Drought-response-strains.

## Results

### Strain-level drought survival

Following the single 6 h drought exposure (D1), all but three strains (*Rhodoferax* sp., *Iodobacter* sp., *Undibacterium* sp.) were able to resume growth upon rewetting (Supplementary Figure 2). Cell viability of the 19 surviving strains ranged from no detectable growth to 1 × 10^8^ cells/ml (average 6 × 10^6^ cells/ml), immediately after resuspension. The mismatch between survival and no detectable growth on agar plates can be explained by the dilution effect from a low cell number being transferred (only 6 μL). After repeated drought exposure (D4), four additional strains failed to recover (*Telluria* sp., *Flavobacterium* sp., *Janthinobacterium* sp., and *Pseudomonas kulmbachensis;* Supplementary Figure 2), and the 15 surviving stains ranged again between zero and 1 × 10^8^ viable cells/ml (average 2 × 10^7^ cells/ml).

### Drought-induced changes in growth traits

**Figure 2:**
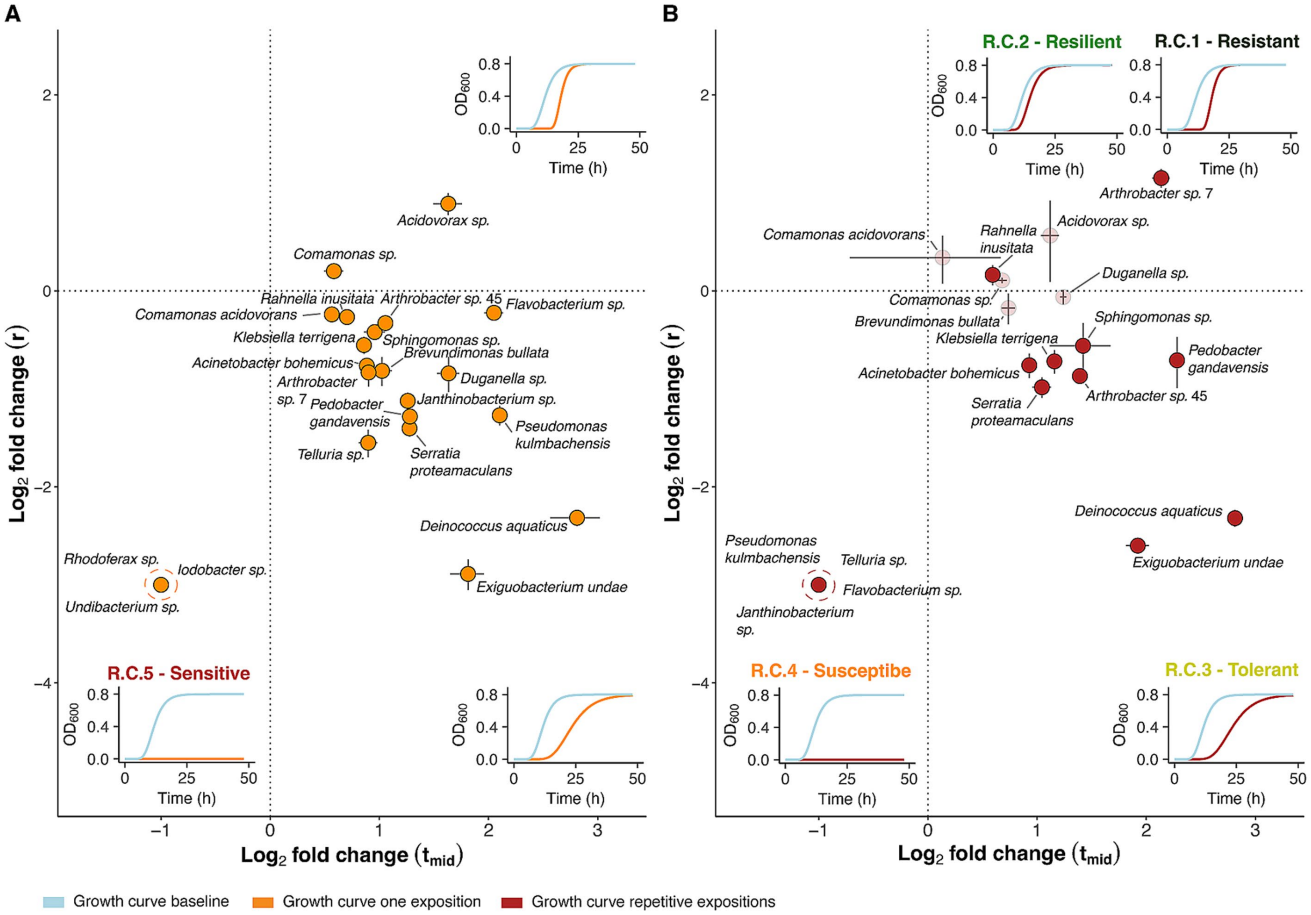
Changes in growth traits of glacier-fed stream bacterial strains following drought exposure. **(A)** Log_2_ fold change in growth rate (*r*, *y*-axis) and lag-phase (*t_mid_*, *x*-axis) between the single drought exposure (D1) and the baseline condition (D0), for each strain. **(B)** Log_2_ fold change in *r* and *t_mid_* between the repeated drought exposure (D4) and the baseline (D0), for each strain. Positive log_2_ fold change values for *t_mid_* indicate an increase in lag-phase after treatment, while positive value in *r* indicate an increase in growth rate. Shaded points denote strains from which *r* did not differ significantly from the baseline. Strains with negative fold changes values in both *r* and *t_mid_* correspond to bounded values, representing strains that did not grow after drought exposure. Response category (R. C.) indicates the strains’ drought response classification, ranging from resistant to sensitive (Figure 1), and are illustrated by a representative growth curve. *r*: Growth rate. *t_mid_*: Time to reach half of the carrying capacity (lag-phase).

Independently of drought treatment (D1, D4), the regrowing populations of all surviving strains, except *Comamonas acidovorans*, showed significantly longer (Wilcoxon; *p* < 0.05) lag phase durations (*t*_mid_) than compared to the pre-drought condition (D0) (Figure 2). This increase in lag phase can be explained in part by the lower number of viable cells transferred prior to drying (*R*^2^ = 0.21, *p* < 0.001, Supplementary Figure 3) and by drought-induced mortality, as only 6 μL of culture were used.

Similarly, after exposure to a single drought (D1), all surviving strains exhibited significantly reduced growth rate (r) compared to pre-drought values (Wilcoxon; *p* < 0.05), except for *Acidovorax* sp. and *Comamonas* sp. which increased *r* (Figure 2A). After repeated drought exposure (D4), however, *Comamonas acidovorans*, *Comamonas* sp., *Brevundimonas bullata*, *Duganella* sp. and *Acidovorax* sp. increased their growth rate and regained baseline growth (Figure 2B). Interestingly, *Arthrobacter* sp. 7 and *Rahnella inusitata* had significantly higher *r* (Wilcoxon; *p* < 0.05), whereas all other strains remained with reduced *r* (Figure 2B): *Sphingomonas* sp., *Pedobacter gandavensis*, *Klebsiella terrigena*, *Acinetobacter bohemicus*, *Serratia proteamaculans*, *Deinococcus aquaticus*, and *Exiguobacterium undae*.

### Drought response categories

**Figure 3:**
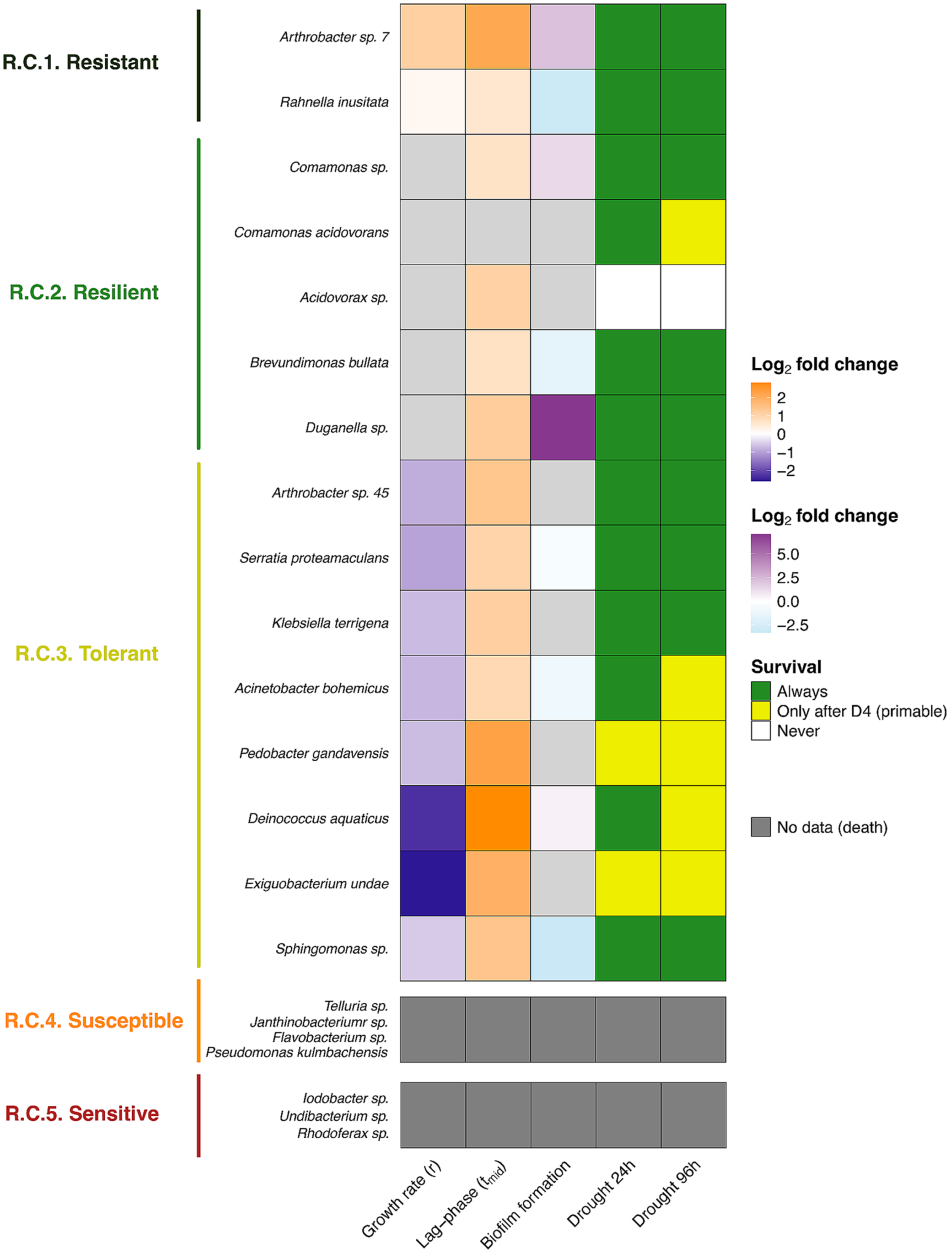
Growth trait changes in response to repeated drought and survival under extended drought periods of glacier-fed stream bacterial strains. Heatmap summarizing strain-specific changes in growth traits between the repeated drought (D4) and baseline (D0) conditions, alongside their survival to extended drought periods (24 h and 96 h). Columns represent log_2_ fold change values in *r*, *t_mid_* and biofilm formation between D4 and D0, as well as categorical survival to 24 h and 96 h droughts. Color scales indicate the magnitude and direction of growth traits changes (light gray = no significant change) or survival status (indicating whether strains exhibit a priming response, defined as the ability to survive extended drought only after prior exposure, but not under baseline conditions). Strains are ordered first by drought response category (R. C.) and then by pattern similarity. Strains from R. C. 4 and R. C. 5 did not survive at this stage (no data). *r*: Growth rate. *t_mid_*: Time to reach half of the carrying capacity (lag-phase).

As lag phase duration increased for all except one strain following repeated droughts, we used changes in growth rate between the repeated drought exposure (D4) and the baseline pre-drought (D0) to classify strains into drought response categories (R. C.) (Figure 3): strains exhibiting significantly higher growth rate after repetitive droughts were classified as resistant (R. C. 1), those returning to baseline rate as resilient (R. C. 2), those remaining at a lower growth rate as tolerant (R. C. 3), those unable to regrow from multiple drought exposure as susceptible (R. C. 4), and those that failed to recover from a single drought as sensitive (R. C. 5) (Figure 3). This classification resulted in 2 strains out of 22 considered as resistant, 5 as resilient, 8 as tolerant, 4 as susceptible, and 3 as sensitive (Figure 3).

These response categories were not correlated with biofilm formation, as within each viable category (i.e., R. C. 1 – R. C. 3), some strains significantly (Wilcoxon; *p* < 0.05) increased while others reduced biofilm production (Figure 3). Interestingly, three strains, *Arthrobacter* sp. 7, *Comamonas* sp., and *Duganella* sp., produced biofilm after repeated droughts, whereas they did not under pre-drought conditions. Moreover, the classification of strains into drought response categories was independent of taxonomy, as categories contained strains from diverse orders, and members of the same order were distributed across different response categories. For example, the *Burkholderiales* strains, the most represented order in this experiment (*n* = 9), were assigned across the resilient, the susceptible and the sensitive response categories (R. C. 2, 4 and 5), and the two *Arthrobacter* sp. strains were classified as either resistant or tolerant (R. C. 1 and 3) (Figures 2, 3). This demonstrates high stress responses diversity even across closely related taxa in GFS.

Finally, we exposed the strains to prolonged drought durations (24 h and 96 h) after each treatment (D0, D1, and D4) to further assess their priming potential under more severe stress. While most resistant and resilient strains (R. C. 1 and 2) were able to withstand these longer droughts even without prior drought exposure (D0), *Comamonas acidovorans* was only able to regrow after 96 h of drought following D1 and D4 (Figure 3), and *Acidovorax* sp. survived both 24 h and 96 h drought only after D1, suggesting a priming capability (Supplementary Figure 2). This evidence of priming via the ability to survive severe stress appeared more widespread in tolerant strains (R. C. 3), where half of the strains in this category were able to regrow after the 96 h drought only if they had been previously exposed to 6 h drought events (Figure 3 and Supplementary Figure 2). However, changes in growth rate, lag phase and biofilm formation between D4 and D0 did not differ significantly between strains that consistently survived extended drought and those that acquired survival only after repeated exposure (Wilcoxon; *p* > 0.1).

### Response trajectories across drought exposure

**Figure 4:**
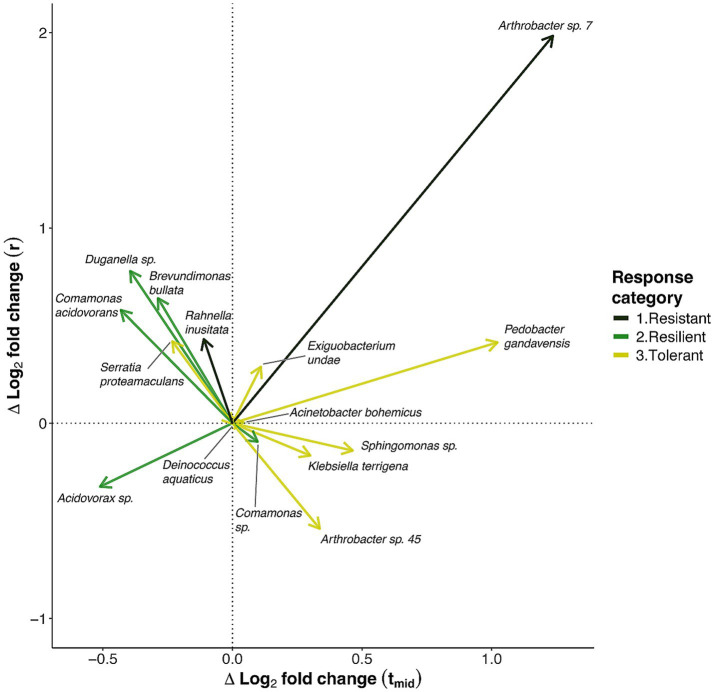
Response trajectories of glacier-fed stream bacterial strains across drought exposure. Arrows represent how each bacterial strain’s growth traits shift between a single drought exposure (D1) and a repeated drought exposure (D4). For each strain, the arrow represents the change in log_2_ fold change values for *t_mid_* (*x*-axis) and *r* (*y*-axis) between these treatments (*Δ* log_2_ fold change), based on data shown in Figure 2. This illustrates strain-specific response trajectories. Positive Δ log_2_ fold change values in *t_mid_* indicate an increase in lag-phase under repeated drought, whereas positive Δ value in *r* indicate an increase in growth rate. Arrows are color-coded by drought response categories. *r*: Growth rate. *t_mid_*: Time to reach half of the carrying capacity (lag-phase).

We next examined changes in growth traits between both drought treatments (D4 vs. D1). Both resistant strains (*Rahnella inusitata* and *Arthrobacter* sp. 7) increased their growth rate with repetitive drought (Figure 4), although they followed divergent trajectories in lag phase duration: *Rahnella inusitata* had a shorter while *Arthrobacter* sp. 7 had a longer lag phase, respectively. Most resilient strains improved at least one growth trait relative to a single drought exposure, typically shortening their lag phase alongside either an increase in *r* (e.g., *Duganella* sp., *Brevundimonas bullata*, *Comamonas acidovorans*), or a decrease in *r* (*Acidovorax* sp.) (Figure 4). Response trajectories within the tolerant category were more variable: two strains showed no additional changes relative to a single drought (e.g., *Deinococcus aquaticus*), whereas others exhibited either improved (3 strains; e.g., *Exiguobacterium undae*) or reduced (3 strains; e.g., *Arthrobacter* sp. 45) growth rates (Figure 4). However, all tolerant strains, except *Serratia proteamaculans*, displayed an increase in lag phase duration with repeated drought (Figure 4).

### Genomic traits linked to drought response

Overall, our strains exhibited genomic features characteristic of bacteria sourced from cryospheric environments (Michoud et al., 2025; Niederdorfer et al., 2017; Bourquin et al., 2022). Genomes were generally constrained in size (3.35–7.02 Mbp, mean 4.94 Mbp), had elevated GC content (36–69%, mean 57.3%) and high 16S rRNA copy numbers (3–11 copies, mean 6 copies) (Table 1).

**Figure 5:**
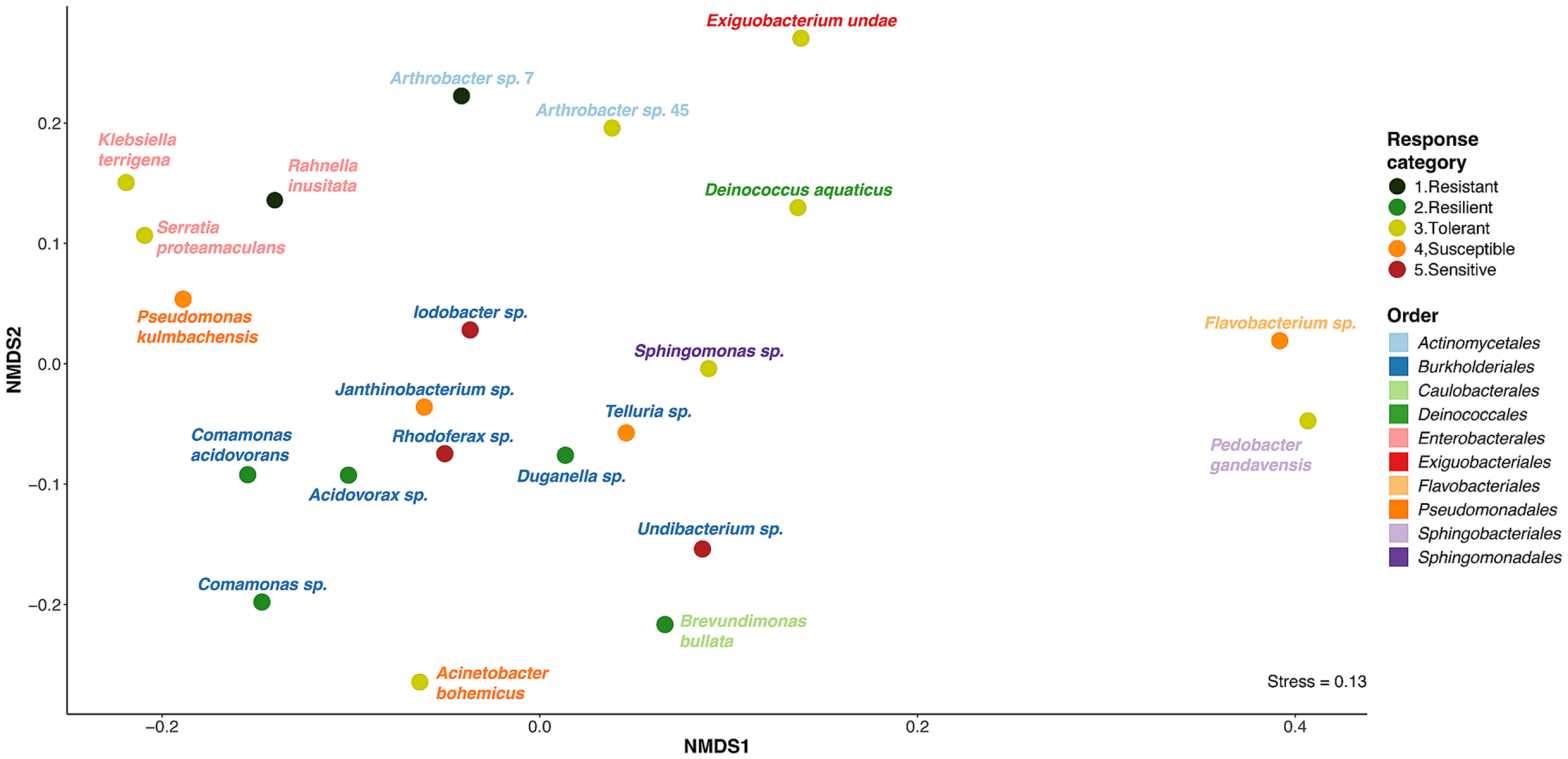
Genomic functional trait similarity among glacier-fed stream bacterial strains. Similarity in genomic functional traits (microTrait granularity level 3) is illustrated by an NMDS ordination based on Bray–Curtis dissimilarity. Each point represents a bacterial strain, is color-coded by its drought response category, and labeled by its taxonomic order.

Exploring similarity in genomic functional traits among the GFS bacterial strains (Figure 5), we found that functional trait composition clustered primarily according to taxonomic order (*R*^2^ = 0.586, *p* < 0.001) and, to a lesser extent, according to response category (*R*^2^ = 0.122, *p* = 0.016). Variance partitioning further confirmed that 37.5% of the variation in functional trait composition was uniquely explained by taxonomic order, whereas 4.5% was uniquely explained by drought response category.

**Figure 6:**
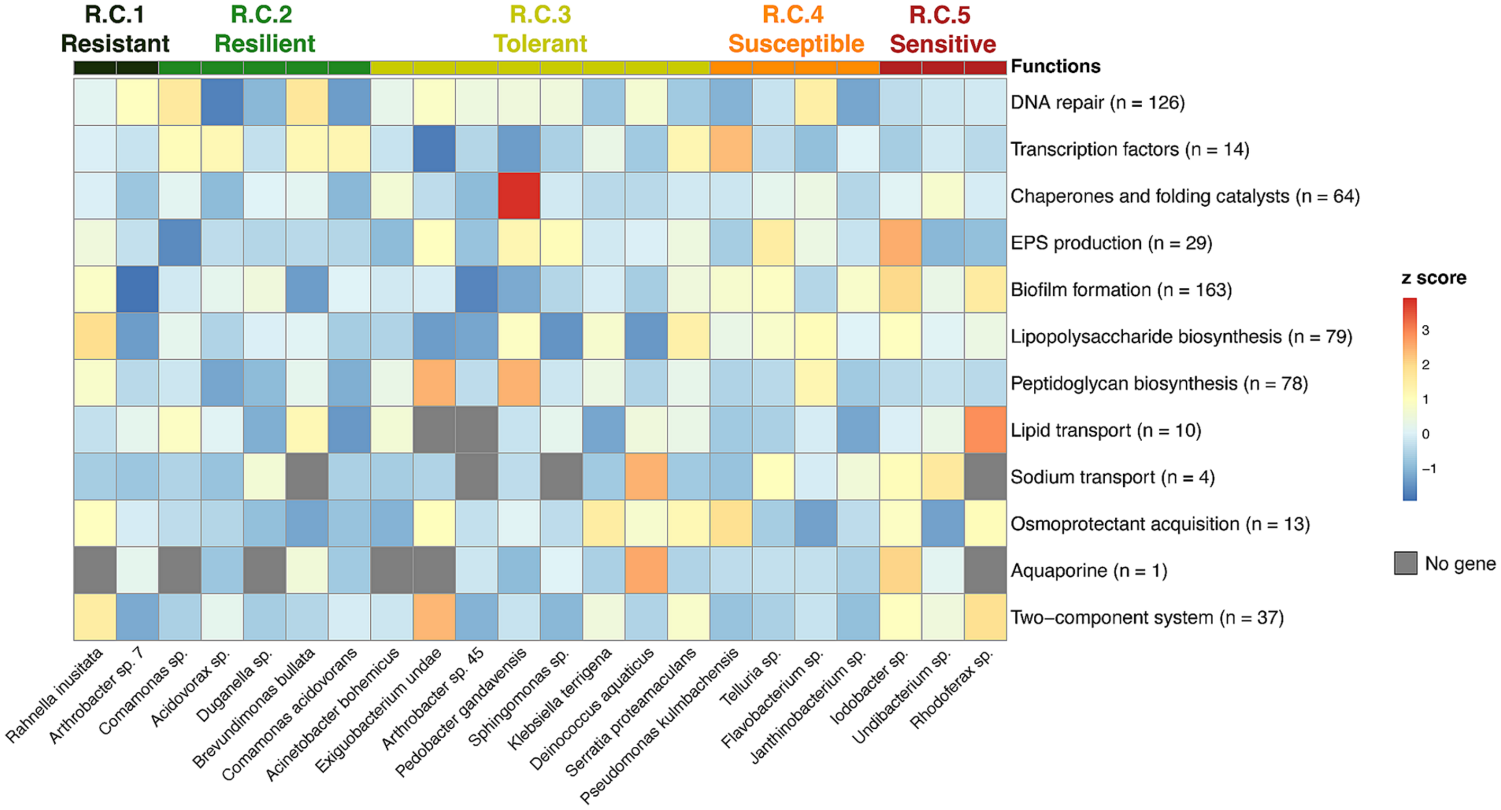
Differential functional profiles of genes associated with drought-stress response across individual glacier-fed stream strains. For each strain, functional profiles of genes associated with drought-stress were normalized by the total number of genes in their genome, and subsequently z-transformed to facilitate comparison of relative investment in drought-response functions among strains. *n* indicates the number of KEGG orthologs (KOs) assigned to each functional category. Strains are ordered by drought response category.

Independent of the drought response category, all strains possessed genes associated with known drought-stress response pathways but showed clear differences in the relative number of these genes in individual functional categories (Figure 6). Despite being isolated from natural biofilms, only six out of the 22 strains formed detectable biofilms under baseline conditions, prior to any drought exposure (Table 1). All strains, however, contained genes associated with biofilm formation or EPS production (Figure 6), but the number of these genes did not correlate with the amount of biofilm produced. All strains also had transporters for osmoprotectants and compatible solutes. After double-standardization, distinct functional signatures emerged across strains (Figure 6). General stress-related functions such as chaperones varied greatly across the strains, whereas DNA repair functions appeared more represented in the genomes of the resistant and tolerant strains, and transcription factors were more abundant in genomes of resilient strains (Figure 6). In contrast, biofilm formation and membrane-related functions, including lipopolysaccharide and lipid transport, were more prominent in the genomes of susceptible and sensitive strains (R. C. 4 and 5, Figure 6). KOs associated with osmoprotectants acquisition and transmembrane transport (sodium transporters and aquaporins) were comparatively less represented in resistant and resilient strains, despite their relevance to drought response. Despite these overall trends, the relative importance of drought-stress associated genomic features still varied among strains of the same response category.

## Discussion

As climate change is expected to increase the frequency and intensity of environmental disturbances, understanding microbial strategies for coping with stress has become increasingly important. In GFSs, for example, droughts are projected to occur more frequently (Paillex et al., 2020; Brunner et al., 2023); however, it remains unclear how individual GFS bacteria tolerate or resist desiccation stress. Our drought-exposure experiment provides a strain-level perspective on the diversity of drought response in environmental bacteria. Moreover, the experimental design allowed us to assess whether repeated drought events act as a selective filter or, conversely, induce stress priming in GFS biofilm bacterial strains. Surprisingly, drought sensitivity was common (7 out of 22 strains), despite natural hydrological fluctuations in GFSs (Smith et al., 2001; Hannah et al., 2007) and the periodic exposure of these ecosystems to drying conditions (Malard et al., 2006; Llanos-Paez et al., 2025). Nonetheless, most GFS biofilm strains survived desiccation and displayed a broad spectrum of drought responses (resistance, resilience and tolerance), highlighting the importance of drought adaptive traits for GFS bacteria. Importantly, these response patterns could not be explained solely by taxonomy or by genomic traits. These results demonstrate that even closely related bacteria vary substantially in their stress responses and suggest that drought poses a significant physiological challenge for microorganisms inhabiting GFSs.

The wide range of drought-induced growth responses observed among our strains highlights the importance of response diversity for coping with desiccation in GFS biofilm bacteria. These differences appeared largely independent of phylogeny, as strains within the same taxonomic group displayed different stress responses. For example, members of the *Burkholderiales* included both resilient (e.g., *Acidovorax* sp., *Comamonas* sp.) and sensitive taxa (e.g., *Undibacterium* sp., *Rhodoferax* sp.) (Figure 2). Even two *Arthrobacter* sp. strains diverged in their responses, with one classified as resistant and the other as tolerant to drought. Such absence of links between phylogeny and stress response phenotype has also been reported in soil bacteria and fungi exposed to desiccation and moisture stress, respectively (Alster et al., 2021; Scales et al., 2023), reinforcing that closely related environmental microorganisms can vary substantially in their response strategies, although response diversity may emerge at a finer taxonomic level than genus, or in a population only.

At the community level, response diversity is thought to stabilize the microbial functional responses and enhance bacterial survival in fluctuating environments (Beier et al., 2015; Girard et al., 2019; Burton et al., 2022). Interestingly, the drought responses observed in our individual strains were partially consistent with patterns detected in a recent field experiment that focused on community-level drought response (Touchette et al., 2025). Specifically, the genera *Sphingomonas* and *Deinococcus*, both of which were drought-tolerant in our present study (Figure 2), were favored in microbial communities exposed to drought (Touchette et al., 2025). However, *Telluria* (referred to as *Massilia* in some databases), although enriched in the community-level study, did not survive multiple droughts when tested individually (Figure 2). This highlights the limitation of scaling single-isolate responses to whole-community dynamics and reinforces the benefit of a communal lifestyle in natural biofilm communities (Flemming et al., 2016).

Survival to drought came at a cost in our bacterial strains: aside from *Acidovorax* sp. and *Comamonas* sp., all surviving strains showed decreased growth rate and increased lag phase after a single drought event (D1, Figure 2A), suggesting decreased fitness. Such growth-survival trade-offs are ecologically important because rapid growth is critical for success in highly dynamic ecosystems, as in GFSs, where cells can take advantage of favorable conditions to grow quickly (Geisel et al., 2011). Yet, slower growth can inherently enhance stress response by shifting cellular investment toward protective functions, thereby improving survival following disturbances (Zhu and Dai, 2024). After repeated drought exposure (D4), however, strain responses began to diverge, indicating that bacteria adopted different strategies to cope with recurrent stress. Some strains regained baseline or improved their growth rates (Figure 2B), suggesting acclimation to repeated drought, whereas others maintained reduced growth (Figure 2B), consistent with an investment in stress protection at the expense of proliferation. In contrast, strains that failed to recover (Figure 2B) likely lacked the physiological capacity to withstand repeated drying. Moreover, the prolonged lag phases observed in nearly all surviving strains after repeated drought (Figure 4) may represent a cautious recovery strategy that delays growth until conditions are favorable (Kolter et al., 2022), although they may partly reflect an additional dilution effect associated with reduced viable cells transferred after each drying/rewetting cycle (Supplementary Figure 3).

Moreover, the strain response categories showed various response trajectories (Figure 4), with growth traits either improving or worsening between single and repeated droughts. For example, half of the strains exhibited progressively longer lag phases between D1 and D4 (Figure 4). Prolonged lag phase has been shown to enhance stress tolerance in *E. coli*, and theory predicts that selection for both rapid growth and stress survival can lead to broad lag phase distributions within populations (Moreno-Gámez et al., 2020). Such delayed growth can also emerge from bet-hedging strategies, where only part of the population resumes rapid growth while others remain in a stress-protected state (Solopova et al., 2014). However, these patterns may also be influenced by the progressive reduction in viable cells transferred after each drying/rewetting cycle, which explained 21% of the observed variation (Supplementary Figure 3). Whether this reflects an experimental constraint, an ecological consequence of drought-induced mortality, or a combination of both remains unresolved. In contrast, several strains showed shorter lag phases between a single to repeated drought exposures (Figure 4). This pattern may reflect the enrichment of drought-robust cells during sequential transfers, where better-suited cells resume growth more rapidly and gradually dominate the population, rather than a shift in physiology. Moreover, half of the strains increased their growth rate when comparing D1 to D4 (Figure 4). Growth metabolism models show that bacteria balance resource allocation between stress protection and overall biosynthesis and growth (Scott et al., 2010; Ramin and Allison, 2019). If repeated drought reduces the need for costly protective investment as surviving cells are better suited for stress response, resources can be redirected toward faster growth. In contrast, other strains showed reduced growth rates, consistent with a need to invest in stress-protective functions, limiting rapid proliferation (Zhu and Dai, 2024). However, future work would be necessary to disentangle which dominant mechanism (physiological regulation vs. population filtering) is at play. For example, monitoring changes in the proportion of viable versus non-viable cells across drought cycles could clarify the extent to which selection over repeated droughts contributes to the observed patterns in lag phase and growth rate.

The fact that roughly half of our strains recovered their growth traits after repeated droughts or even improved them relative to their initial post-drought response, implies that some level of strain-specific stress priming occurred in response to recurring environmental disturbances. This is further supported by the acquired ability of five strains to survive longer drought durations (24 h and/or 96 h) only after a prior exposure to a short drought (Figure 3). This is consistent with observations of drought adaptation in GFS biofilm communities, where drought-adapted taxa were favored and bacterial metabolism was stimulated under repeated droughts (Touchette et al., 2025, 2026), although such effects had not yet been demonstrated at the level of individual GFS bacterial strains. While drought priming is well documented at community scale, particularly in soils (Leizeaga et al., 2022; Canarini et al., 2021; Andrade-Linares et al., 2016; Rillig et al., 2015), explicit demonstration at the level of individual bacterial strains remains scarce, especially in freshwater ecosystems. Therefore, our results provide a rare evidence of history-dependent stress tolerance in freshwater bacteria (e.g., Mathis and Ackermann, 2016), reinforcing that priming is not just an emergent community-level property, but plays a role at the level of individual population too.

Considering that our 22 bacterial strains were isolated from GFS biofilms, we expected that stimulation of biofilm formation through EPS accumulation would represent a major strategy to withstand drought stress, a mechanism well documented at the biofilm community level (Romaní et al., 2025; Sabater et al., 2016; Flemming and Wingender, 2010). However, despite all 22 strains encoding genes associated with EPS synthesis (Figure 6), only six strains in our experiment produced biofilm under baseline conditions (Table 1), which were either drought-resistant, resilient or tolerant (Table 1). Moreover, only four strains increased biofilm production after repetitive droughts (Figure 3). This mismatch between genomic potential and phenotype suggests that biofilm formation in many taxa may depend on interspecies interactions, cross-feeding, quorum sensing or other environmental signals (Sadiq et al., 2023; Elias and Banin, 2012). In natural biofilms, such interactions among community members can modify biofilm development and stress responses, generating community-level traits that are absent or weaker in monoculture, as previously supported by the synergistic biofilm formation observed in some co-cultures of our GFS isolates (Gonzalez Mateu et al., 2026). Alternatively, this mismatch may be an artifact of the use of a relatively rich culture medium in our experiment. Notably, the higher proportion of biofilm and membrane related genes in susceptible and sensitive strains (Figure 6) is counterintuitive and may indicate that these taxa are capable of biofilm formation but do not express these traits under our specific growth conditions or reinforce the idea that biofilm-mediated drought protection may primarily emerge at community-level rather than as an individual response.

Finally, although environmental pressures can select for resistance-associated genes that shape the bacterial genome and provide a form of stress memory (Scanlon et al., 2025), the variation in genomic functional traits only weakly explained the drought response categories in our study. As expected, genomic trait variation was largely structured by taxonomy and all strains encoded genes associated with general stress response, such as DNA repair, membrane modification, regulation of chaperones, heat shock proteins, and specific transcription factors (Guan et al., 2017), as well as genes linked specifically to drought and osmotic stress, including biofilm/EPS production (Flemming and Wingender, 2010; Sabater et al., 2016), osmoprotectants and compatible solutes acquisition, and osmotic sensing (Kunte, 2006). Not unexpectedly, the proportion of these genes in the genome varied among the strains (Figure 6). Overall, our results suggest that modes of response to drought and priming are highly strain-specific and cannot be predicted based on the presence of stress-related genes. Likely, their regulation via (post)transcriptional, (post)translational and epigenetic controls (Scanlon et al., 2025; Andrade-Linares et al., 2016) play a dominant role in their drought adaptation, as evidenced by the production of biofilm in some strains only after prior exposure to drought (Table 1 and Figure 3). Another possible explanation is that GFS bacteria are simultaneously exposed to multiple stressors in their natural environment, such as temperature fluctuations and abrasion by suspended sediments (Uehlinger et al., 2010; Boix Canadell et al., 2021). These co-occurring pressures may favor broad stress-response systems rather than drought-specific mechanisms.

## Conclusion

GFS biofilm bacteria exhibit a broad capacity to respond to drought, but often at the cost of altered growth dynamics. When exposed to repeated droughts, strain response diverged markedly, ranging from direct stimulation or reduction of growth, gradual recovery to mortality. Evidence for stress priming among several strains, expressed as improved growth rate with repeated drought exposure and as survival to prolonged drought only after a prior exposure, suggests that microbial memory-like processes operate at the level of individual populations. Since these drought adaptation and priming capacities were overall rather independent from taxonomy or genomic functional traits, our findings suggest that regulatory mechanisms, rather than functional potential alone, are key determinants of drought tolerance. Therefore, future studies combining comparative genomics with transcriptomic analyses during drought exposure would be needed to identify the regulatory pathways underlying these responses. Our strain-level heterogeneity emphasizes that GFS biofilm communities harbor diverse stress-response strategies, and that drought resilience at the ecosystem level likely emerges from the integration of these varied individual responses.

## Data Availability

The data presented in this study can be found in the NCBI (https://www.ncbi.nlm.nih.gov/), under BioProject accession PRJNA1171349.
