# Supplementary material for: Diverse drought responses and evidence for stress priming in glacier-fed stream bacteria
Source: Front Microbiol. 2026 Aug 5;17:1898462. doi: 10.3389/fmicb.2026.1898462 (PMC13486165; doi:10.3389/fmicb.2026.1898462)
Supplement: Supplementary file 1 [file Supplementary_file_1.DOCX]

**Supplementary Figure 1. Baseline, D1 and D4 growth curves of the 22 glacier-fed stream bacterial strains.**

Growth curves used for growth trait analyses, shown per treatments and per strain, after subtracting the highest negative control value and removing wells with abnormal behavior. D0: baseline, D1: one drought exposure, D4: repeated drought exposure.

**Supplementary Figure 2. Glacier-fed stream bacterial strain survival to drought exposure.**

Categorical survival of each bacterial strain after 24 h or 96 h drought, either without prior 6 h drought exposure (D0), after one 6 h drought exposure (D1), or after four consecutive 6 h drought/recovery cycles (D4).

**Supplementary Figure 3. Linear relationship between cell viability and t_mid_.**

This negative linear relationship indicates that t_mid_, a proxy for lag phase duration, increases as the initial number of viable cells decreases. The x-axis is presented a log_10_ scale.

t_mid_: time to reach half of the carrying capacity.

**Supplementary Table 1. Complete taxonomic classification of the used GFS bacterial strains, based on GTDB-Tk v.2.2.2.**

| Strain # | Phyla | Class | Order | Family | Genus | Species | NCBI  Accession # |
| --- | --- | --- | --- | --- | --- | --- | --- |
| 1 | *Pseudomonadota* | *Gammaproteobacteria* | *Burkholderiales* | *Chitinibacteraceae* | *Iodobacter* | *sp.* | SAMN53262576 |
| 2 | *Pseudomonadota* | *Alphaproteobacteria* | *Sphingomonadales* | *Sphingomonadaceae* | *Sphingomonas* | *sp.* | SAMN53262585 |
| 3 | *Actinomycetota* | *Actinomycetes* | *Actinomycetales* | *Micrococcaceae* | *Arthrobacter* | *sp.* | SAMN53262568 |
| 4 | *Pseudomonadota* | *Gammaproteobacteria* | *Burkholderiales* | *Burkholderiaceae* | *Comamonas* | *sp.* | SAMN53262570 |
| 5 | *Actinomycetota* | *Actinomycetes* | *Actinomycetales* | *Micrococcaceae* | *Arthrobacter* | *sp* | SAMN53262567 |
| 6 | *Pseudomonadota* | *Gammaproteobacteria* | *Burkholderiales* | *Burkholderiaceae* | *Janthinobacterium* | *sp.* | SAMN53262577 |
| 7 | *Pseudomonadota* | *Gammaproteobacteria* | *Burkholderiales* | *Burkholderiaceae* | *Comamonas* | *acidovorans* | SAMN53262571 |
| 8 | *Deinococcota* | *Deinococci* | *Deinococcales* | *Deinococcaceae* | *Deinococcus* | *aquaticus* | SAMN53262572 |
| 9 | *Pseudomonadota* | *Gammaproteobacteria* | *Enterobacterales* | *Enterobacteriaceae* | *Rahnella* | *inusitata* | SAMN53262582 |
| 10 | *Pseudomonadota* | *Gammaproteobacteria* | *Burkholderiales* | *Burkholderiaceae* | *Telluria* | *sp.* | SAMN53262579 |
| 11 | *Pseudomonadota* | *Gammaproteobacteria* | *Pseudomonadales* | *Moraxellaceae* | *Acinetobacter* | *bohemicus* | SAMN53262566 |
| 12 | *Pseudomonadota* | *Gammaproteobacteria* | *Enterobacterales* | *Enterobacteriaceae* | *Klebsiella* | *terrigena* | SAMN53262578 |
| 13 | *Pseudomonadota* | *Alphaproteobacteria* | *Caulobacterales* | *Caulobacteraceae* | *Brevundimonas* | *bullata* | SAMN53262569 |
| 14 | *Bacteroidota* | *Bacteroidia* | *Sphingobacteriales* | *Sphingobacteriaceae* | *Pedobacter* | *gandavensis* | SAMN53262580 |
| 15 | *Pseudomonadota* | *Gammaproteobacteria* | *Burkholderiales* | *Burkholderiaceae* | *Duganella* | *sp.* | SAMN53262573 |
| 16 | *Pseudomonadota* | *Gammaproteobacteria* | *Enterobacterales* | *Enterobacteriaceae* | *Serratia* | *proteamaculans* | SAMN53262584 |
| 17 | *Pseudomonadota* | *Gammaproteobacteria* | *Burkholderiales* | *Burkholderiaceae* | *Acidovorax* | *sp* | SAMN53262565 |
| 18 | *Pseudomonadota* | *Gammaproteobacteria* | *Burkholderiales* | *Burkholderiaceae* | *Undibacterium* | *sp.* | SAMN53262586 |
| 19 | *Bacillota* | *Bacilli* | *Exiguobacteriales* | *Exiguobacteriaceae* | *Exiguobacterium* | *undae* | SAMN53262574 |
| 20 | *Pseudomonadota* | *Gammaproteobacteria* | *Pseudomonadales* | *Pseudomonadaceae* | *Pseudomonas* | *kulmbachensis* | SAMN53262581 |
| 21 | *Bacteroidota* | *Bacteroidia* | *Flavobacteriales* | *Flavobacteriaceae* | *Flavobacterium* | *sp.* | SAMN53262575 |
| 22 | *Pseudomonadota* | *Gammaproteobacteria* | *Burkholderiales* | *Burkholderiaceae* | *Rhodoferax* | *sp.* | SAMN53262583 |
